# Trends in cesarean section rates in private and public facilities in rural eastern Maharashtra, India from 2010-2017

**DOI:** 10.1371/journal.pone.0256096

**Published:** 2021-08-12

**Authors:** Elizabeth Simmons, Kevin Lane, Sowmya R. Rao, Kunal Kurhe, Archana Patel, Patricia L. Hibberd

**Affiliations:** 1 Department of Global Health, Boston University School of Public health, Boston, MA, United States of America; 2 Department of Environmental Health, Boston University School of Public Health, Boston, MA, United States of America; 3 Lata Medical Research Foundation, Nagpur, Maharashtra, India; 4 Datta Meghe Institute of Medical Sciences, Nagpur, Maharashtra, India; University of Georgia, UNITED STATES

## Abstract

**Introduction:**

Rates of cesarean sections (CS) have increased dramatically over the past two decades in India. This increase has been disproportionately high in private facilities, but little is known about the drivers of the CS rate increase and how they vary over time and geographically.

**Methods:**

Women enrolled in the Nagpur, India site of the Global Network for Women’s and Children’s Health Research Maternal and Neonatal Health Registry, who delivered in a health facility with CS capability were included in this study. The trend in CS rates from 2010 to 2017 in public and private facilities were assessed and displayed by subdistrict. Multivariable generalized estimating equations models were used to assess the association of delivering in private versus public facilities with having a CS, adjusting for known risk factors.

**Results:**

CS rates increased substantially between 2010 and 2017 at both public and private facilities. The odds of having a CS at a private facility were 40% higher than at a public facility after adjusting for other known risk factors. CS rates had unequal spatial distributions at the subdistrict level.

**Discussion:**

Our study findings contribute to the knowledge of increasing CS rates in both public and private facilities in India. Maps of the spatial distribution of subdistrict-based CS rates are helpful in understanding patterns of CS deliveries, but more investigation as to why clusters of high CS rates have formed in warranted.

## Introduction

Cesarean section (CS) is a life-saving procedure when certain complications arise during pregnancy and delivery. At a 1985 meeting of the World Health Organization (WHO), a panel of reproductive health experts identified the “ideal” population-based CS rate to be between 10 and 15% [1]. Since then, global CS rates rose from 12.1% in 2000 to 21.1% in 2015 [2]. This increase prompted WHO to revisit the ideal rate suggested in 1985, and resulted in a recommendation that every effort should be taken to ensure that CS are provided to women in need, rather than focusing on achieving a certain CS rate [3]. In order to avoid major obstetric complications that can lead to maternal and infant death, CS are essential treatment for antepartum hemorrhage, prolonged or obstructed labor, pre-eclampsia or eclampsia, and intrapartum fetal distress [4].

The unprecedented rise in the use of CS in the past two decades can be explained by both an increase of institutional births and increased use of CS within facilities [5]. Reasons for increased CS within facilities are due to factors related to the pregnant women, their families, health professionals and health care systems [6]. Privately owned facilities have disproportionately contributed to increasing CS rates, although rates continue to increase in publicly funded facilities as well [5]. Using the most recent Demographic and Health Survey (DHS) data from 50 low- and middle-income countries between 2000 and 2013, Benova et al. found that privately funded facilities had higher CS rates in every region [7].

Reasons for having CS include both demographic characteristics, such as older maternal age, higher parity and higher socioeconomic status, and clinical indicators, including non-reassuring fetal status, labor arrest disorders, malpresentation, multi-gestation births [8–11]. Consequences of CS include higher maternal and perinatal mortality than with vaginal birth and increased short- and long-term health consequences for mothers, such as uterine rupture, infection or hemorrhage, and infants, including altered immune development, an increased likelihood of allergy, atopy, and asthma, and reduced intestinal gut microbiome diversity [11,12]. There is also an increased risk of maternal death for CS when conducted without a medical indication [13].

Financial schemes to incentivize facility-based deliveries can also influence the rate of CS [14]. In India, Janani Suraksha Yojana (JSY) was implemented in 2005 to reduce maternal and neonatal mortality by promoting institutional delivery as opposed to home deliveries among pregnant women below the poverty line [15]. In the 2005–2006 Indian National Family Health Survey (NFHS-3), 39% of deliveries took place in institutions but in NHFS-4 during 2015–2016, 79% of deliveries occurred in institutions [16,17]. CS rates increased from 9% of all live births in 2005–2006 [16] to 17% in 2015–2016 [17]. The Indian district level household survey in 2011 (DLHS-4) found that CS births were disproportionately high and nearly three times more likely in private facilities than in public facilities [8].

While rates of CS in India have increased in the last 10 years, less is known about the drivers of the change in CS rates and whether they vary over time and geographically. The *Eunice Kennedy Shriver* National Institute of Child Health and Human Development’s (NICHD’s) Global Network (GN), is a multi-site research network representing partnerships of U.S. and international investigators at rural and semi-urban study sites in Guatemala, India (2 sites: Nagpur and Belgaum), Pakistan, Kenya, Zambia and the Democratic Republic of the Congo. The GN Maternal and Newborn Health Registry (MNHR) has been collecting data on a population-based sample of pregnant women and their babies starting in 2008. Using data from the Nagpur, India site, the primary aims of this study are to (1) assess the change in CS rates over time in the public and private facilities where the women deliver their babies and (2) determine the maternal characteristics associated with CS rates in private and public facilities in the catchment area of the study population. A secondary aim of this study was to visualize the geographic distribution of CS rates in the subdistricts over time.

## Materials and methods

### Study population

**Fig 1:**
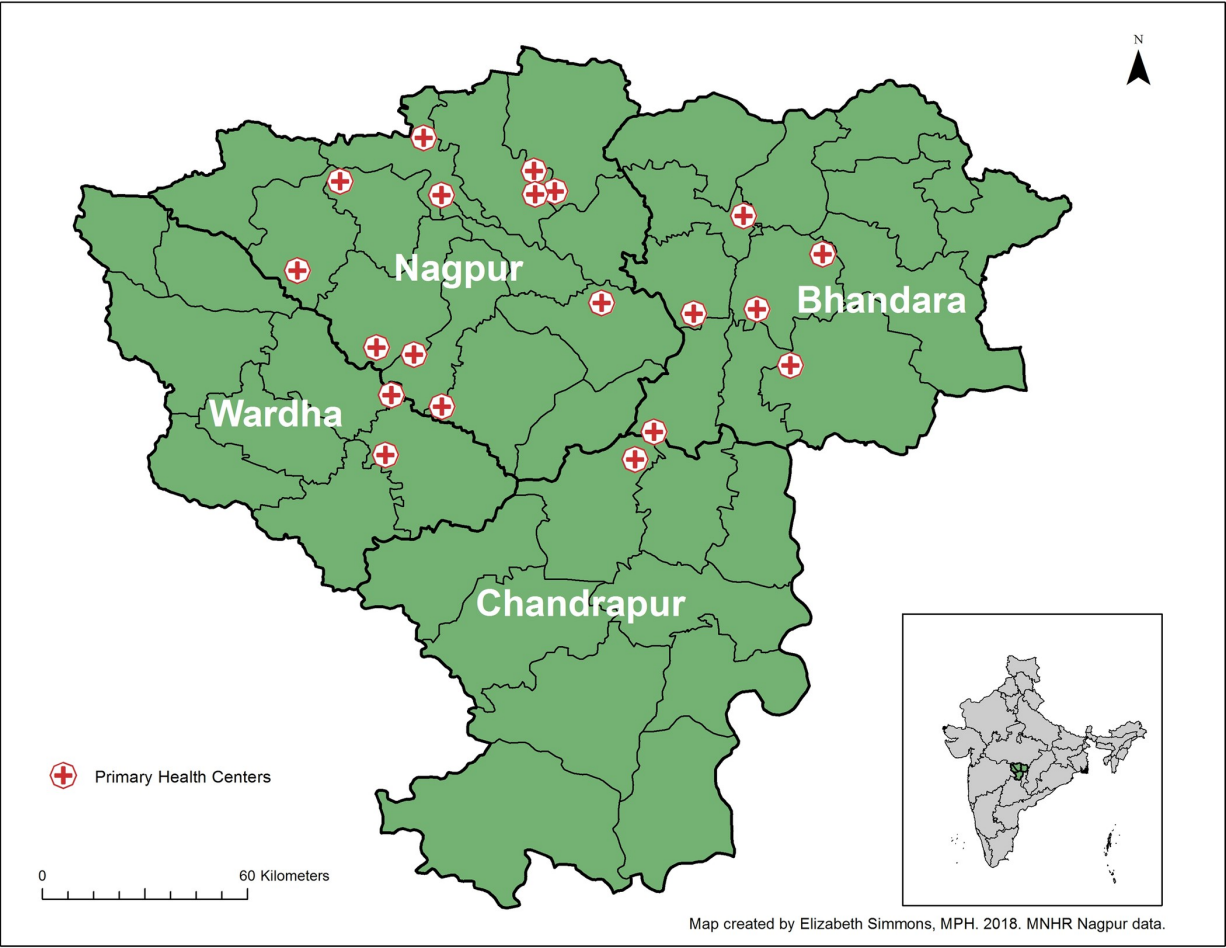
Map of 20 primary health centers in Nagpur area.

Prospectively-collected data from pregnant women and their babies enrolled in the Nagpur site (Eastern Maharashtra) of the MNHR was used for this study. The details of the MNHR registry have been previously published [18]. In brief, each GN site, including the Nagpur site, studies a population of 8 to 20 predetermined geographic areas or clusters. In Nagpur, each cluster is defined as the catchment area of a primary health center (PHC), with 300–500 expected births each year. Between 2010 and 2018, the Nagpur site included 20 PHCs in the Nagpur, Bhandara, Wardha and Chandrapur districts in eastern Maharashtra that extend 25–100 km radially from the urban center, Nagpur city (Fig 1). Women are enrolled as early as possible in pregnancy (usually early in the 2^nd^ trimester of pregnancy, but increasingly over time during the first trimester) and are followed-up after labor and delivery through day 42 post-partum to collect details of maternal and neonatal outcomes.

### Ethical approvals

Pregnant women presenting for antenatal care at one of the Nagpur site’s PHC were informed about the study and invited to participate in the MNH registry. Those who agreed and provided written informed consent were enrolled in the Registry. Institutional Review Boards at Lata Medical Research Foundation (LMRF) and Boston University Medical Campus (BUMC) approved the protocol and consent forms. The GN for Women’s and Child’s Health Research MNMR Study is registered on clinicaltrials.gov under NCT01073475.

### Demographic and health data collection

Three forms were used to collect data on women enrolled in the MNHR [19]. The enrollment form collected background information, residence status and planned delivery location from the mother at the time of enrollment. The enrollment form collected maternal age (<20, 20–24, 25–20, 30+ years), parity (≥1 vs. 0), maternal education (None, 1–6, 7–12, >12 years) and anemia [yes (hemoglobin < 11 g/dL) vs. no (hemoglobin ≥ 11 g/dL)]. Within 7 days of delivery, the perinatal form was completed with data on health care services the mother received during pregnancy, delivery information, neonatal and maternal outcomes and treatments provided at time of delivery. These data were used to derive the gestational age at birth (full term (≥37 weeks) vs. preterm), birth weight (normal (≥2500 g) vs. low) and year of delivery (2010–2017). The perinatal form also collected information on our primary outcome, mode of delivery (vaginal/vaginal assisted versus cesarean section) for the women enrolled in the MNHR.

### Identification and geocoding of public and private facilities with CS capability

Between 2010 to 2017, women participating in the Nagpur site of MNH Registry delivered at 133 facilities (101 private; 32 public) with CS capabilities. For the purposes of this study, having CS capability was defined as being a secondary level facility or higher, as primary health centers manage normal deliveries only [20]. While it would have been ideal to identify facilities with a blood bank on site, this information was not readily available in this study, particularly for the private facilities. In the public health system, we are confident that secondary facilities and higher have the ability to conduct CS. We kept this definition consistent for private facilities in the absence of more information. During field research officer visits to each facility, the GPS coordinates were collected at the front gate of the facility using a smartphone. The number of private facilities used by women in the MNHR in the area more than tripled from 2010 to 2017, from 29 to 101. Growth in public facilities in the area increased from 22 in 2010 to 32 in 2017.

### Statistical methods

Descriptive statistics were calculated for all variables to describe the study population. Chi-square analysis was used to compare differences in proportions between public and private facilities. CS rates of each subregion were calculated for each year from 2011 to 2017 and displayed geographically using ArcGIS (Esri, Redlands, California). We obtained Odds ratios (OR) and 95% Confidence Intervals (CI) from multivariable generalized estimating equations models that assessed the association of public/private facilities with having a CS adjusting for known risk factors. We also tested the interaction between maternal age and parity was included in the multivariable model. We tested whether the trend in the odds of having a CS increased significantly with each year by including the delivery year as a continuous variable in the models. We further used a random effects model with random intercept and slope to assess if the trajectory of the CS rates was similar in public and private facilities. This accounts for the within-facility and between-facility variability. All analyses were conducted using SAS 9.4 (SAS Institute, Cary, NC) and a two-sided p<0.05 was considered to be significant.

## Results

**Fig 2:**
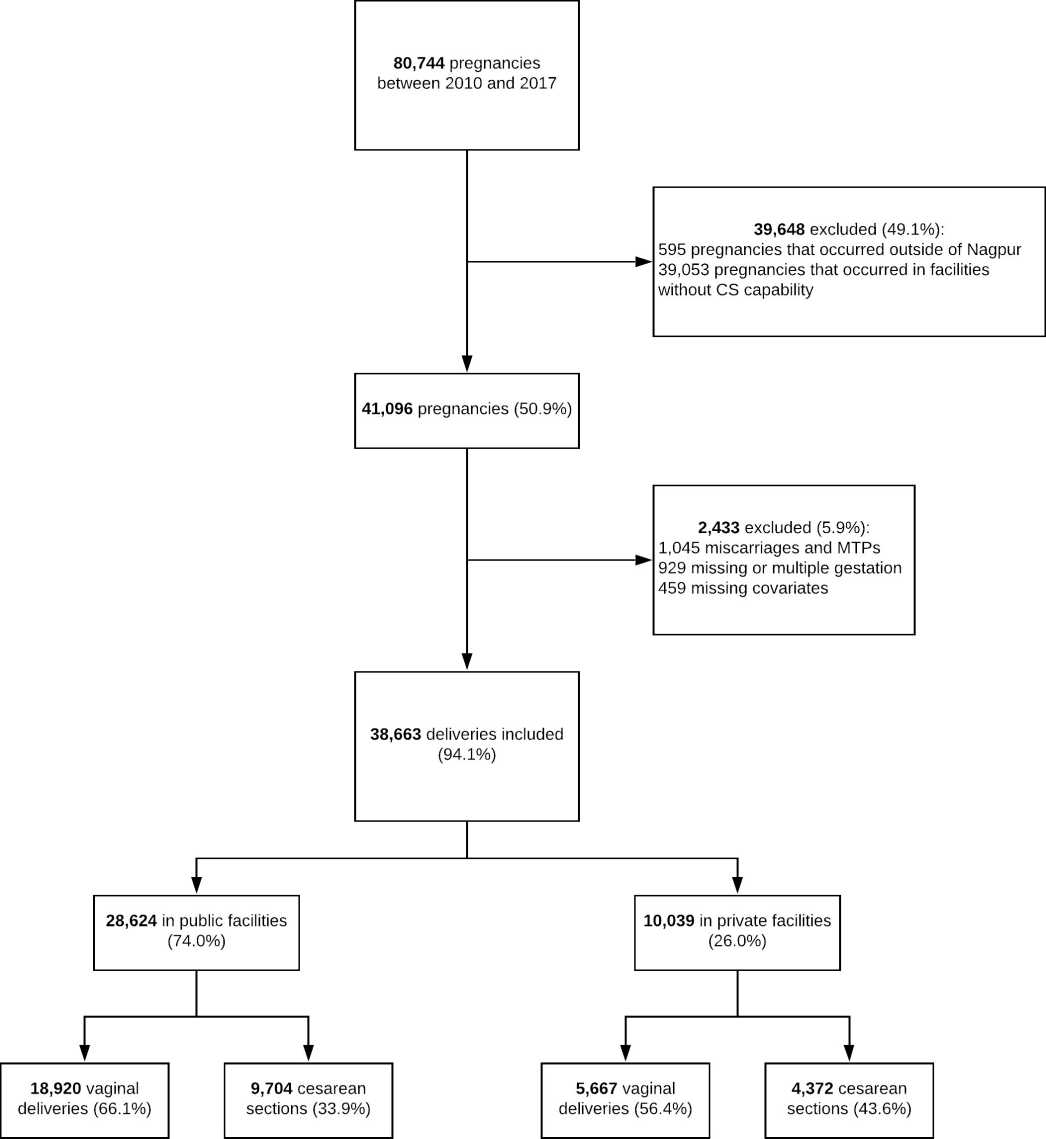
Flow diagram.

From 2010 to 2017, 80,744 deliveries were recorded in the MNHR. This analysis included the 38,663 deliveries that occurred in facilities where CS were performed and excluded women who had a miscarriage or a medical termination of pregnancy (1,045), women with a multiple or missing gestation (708), and women with missing covariates (136). Thirty-six percent of all MNHR registry deliveries, 34% of all MNHR deliveries in public facilities and 44% of all MNHR deliveries in private facilities were by CS (Fig 2).

Table 1 displays characteristics of our study sample by delivery facility type. Over half of women age 30 and over who delivered in private facilities had a CS, compared with only 43.5% of women in the same age group who delivered in public facilities. Almost half of women with more than 12 years of education who delivered in a private facility had a CS whereas 37.9% of women with the same educational attainment had a CS in public facilities.

**Table 1:** Background characteristics of population by type of facility of delivery.

	Type of facility								
	All deliveries n = 38663			All Public Deliveries n = 28624			All Private Deliveries n = 10039		
	CS	No CS	p^a^	CS	No CS	p^a^	CS	No CS	p^a^
	N(Row%)	N(Row%)		N(Row%)	N(Row%)		N(Row%)	N(Row%)	
Maternal Age									
< 20 years	257 (28.9)	633 (71.1)	< .0001	197 (27.7)	514 (72.3)	< .0001	60 (33.5)	119 (66.5)	< .0001
20–24 years	8563 (34.2)	16505 (65.8)		6049 (32.1)	12770 (67.9)		2514 (40.2)	3735 (59.8)	
25–29 years	4336 (40.2)	6450 (59.8)		2874 (37.1)	4878 (62.9)		1462 (48.2)	1572 (51.8)	
30+ years	920 (47.9)	999 (52.1)		584 (43.5)	758 (56.5)		336 (58.2)	241 (41.8)	
Parity									
≥1	6099 (36.4)	10652 (63.6)	0.99	4234 (33.9)	8253 (66.1)	0.99	1865 (43.7)	2399 (56.3)	0.74
0	7977 (36.4)	13935 (63.6)		5470 (33.9)	10667 (66.1)		2507 (43.4)	3268 (56.6)	
Education									
None	244 (28.3)	618 (71.7)	< .0001	175 (27.3)	466 (72.7)	< .0001	69 (31.2)	152 (68.8)	< .0001
1–6 years	1087 (29.3)	2629 (70.8)		847 (27.9)	2186 (72.1)		240 (35.1)	443 (64.9)	
7–12 years	9621 (36.0)	17072 (64.0)		6912 (34.1)	13365 (65.9)		2709 (42.2)	3707 (57.8)	
>12 years	3124 (42.3)	4268 (57.7)		1770 (37.9)	2903 (62.1)		1354 (49.8)	1365 (50.2)	
Gestational Age									
Full term	12978 (36.9)	22236 (63.2)	< .0001	9022 (34.5)	17105 (65.5)	< .0001	5131 (56.5)	3956 (43.5)	0.92
Preterm	1098 (31.8)	2351 (68.2)		682 (27.3)	1815 (72.7)		536 (56.3)	416 (43.7)	
Birth weight									
Normal	9045 (37.9)	14831 (62.1)	< .0001	6120 (35.5)	11133 (64.5)	< .0001	2925 (44.2)	3698 (55.8)	0.08
Low	5031 (34.0)	9756 (66.0)		3584 (31.5)	7787 (68.5)		1447 (42.4)	1969 (57.6)	
Anemia									
Yes	12433 (35.8)	22344 (64.3)	< .0001	8740 (33.4)	17395 (66.6)	< .0001	3693 (42.7)	4949 (57.3)	< .0001
No	1643 (42.3)	2243 (57.7)		964 (38.7)	1525 (61.3)		679 (48.6)	718 (51.4)	
Year of Delivery									
2010	1253 (28.2)	3196 (71.8)	< .0001	906 (26.3)	2533 (73.7)	< .0001	347 (34.4)	663 (65.6)	< .0001
2011	1388 (30.0)	3236 (70.0)		956 (28.1)	2441 (71.9)		432 (35.2)	795 (64.8)	
2012	1454 (32.8)	2980 (67.2)		1049 (31.0)	2331 (69.0)		405 (38.4)	649 (61.6)	
2013	1706 (34.1)	3300 (65.9)		1192 (31.3)	2623 (68.8)		514 (43.2)	677 (56.8)	
2014	1727 (36.8)	2963 (63.2)		1241 (35.0)	2306 (65.0)		486 (42.5)	657 (57.5)	
2015	2198 (41.9)	3047 (58.1)		1490 (39.5)	2287 (60.6)		708 (48.2)	760 (51.8)	
2016	2257 (43.2)	2972 (56.8)		1481 (40.1)	2209 (59.9)		776 (50.4)	763 (49.6)	
2017	2093 (42.0)	2893 (58.0)		1389 (38.8)	2190 (61.2)		704 (50.0)	703 (50.0)	

^a^ p-value produced using chi-square test of independence.

**Fig 3:**
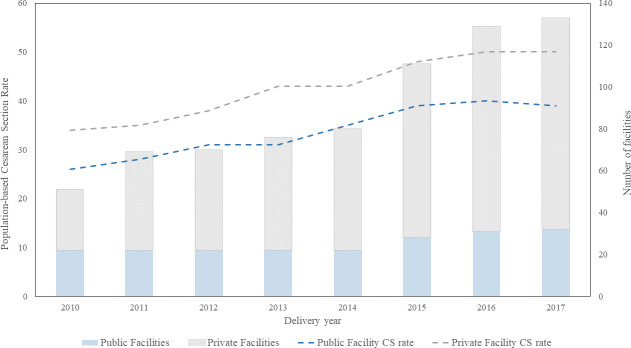
Linear trend in population-based Cesarean section (CS) rate and histogram of number of private and public facilities from 2010 to 2017 by facility type.

**Fig 4:**
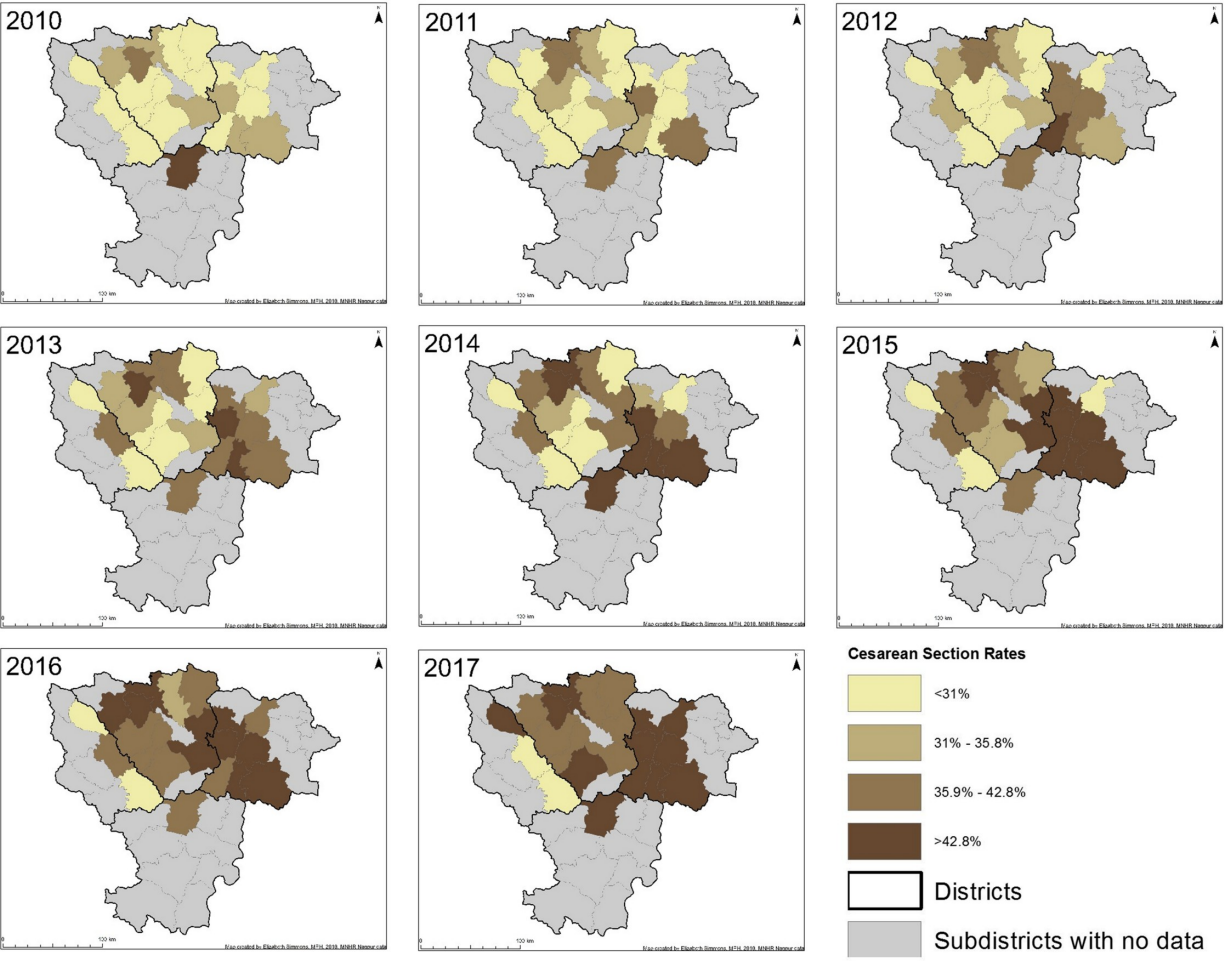
Change in subdistrict population-based cesarean section rate from 2010 to 2017.

The number of private and public facilities and CS rates by type of facility over time are displayed in Fig 3. While the number of public facilities increased slightly over time, the number of private facilities increased by 250% in the 8-year period. However, there was no evidence to suggest that the increase over time was statistically different between private and public facilities over the same period. Subdistrict-based CS rates also increased over time in almost all subdistricts (Fig 4).

Table 2 displays the ORs and 95% CIs evaluating the association between having a CS with type of facility adjusted for known risk factors. Private facilities had a 40% increase in the odds of having a CS delivery compared with public facilities, after adjusting for all covariates [OR(95% CI): 1.40(1.14, 1.72); p<0.01]. The adjusted results indicate the odds of having a CS increased with higher maternal education [1–6 years: 1.28(1.07, 1.54); 7–12 years: 1.50(1.30, 1.72); >12 years: 1.89(1.66, 2.15); p<0.0001], increasing year of delivery [2011: 1.07(0.99, 1.16); 2017: 1.71(1.49, 1.97); p<0.0001] and with being not anemic [1.15(1.05, 1.26); p<0.01]. Nulliparous women at every age had higher odds of delivering by CS than primi- and multiparous women with those aged 30+ having the highest odds [3.24(1.25, 8.41); p<0.0001]. The odds of having a CS decreased for preterm babies [0.82(0.73, 0.91); p<0.001] and for low birth weight babies [0.91(0.86, 0.96); p = 0.001]. The trend test indicated that the odds of having a CS increased by 9% for each year between 2010 and 2017 [1.09(1.08–1.11); p<0.0001].

**Table 2:** Odds ratios (OR) and 95% Confidence Intervals (CI) obtained from multivariable generalized estimating equations models evaluating the association of CS with type of facility adjusting for known risk factors.

	Type of Delivery		Unadjusted Model	p	Adjusted Model^a^	p
	Vaginal Delivery	Cesarean Section				
	n(Row %)		OR (95%CI)		OR (95%CI)	
Type of Facility						
Public (REF)	18920 (66)	9704 (34)		<0.001		<0.01
Private	5667 (56)	4372 (44)	1.50 (1.20, 1.89)		1.40 (1.14, 1.72)	
Maternal Age (years)^b^						
<20 (REF)	633 (71)	257 (29)				
20–24	16505 (66)	8563 (34)				
25–29	6450 (60)	4336 (40)				
30+	999 (52)	920 (48)				
Parity^b^						
≥1 (REF)	10652 (64)	6099 (36)				
0	13935 (64)	7977 (36)				
Maternal age (years), Parity						
<20, ≥1 (REF)	14 (74)	5 (26)		< .0001		< .0001
<20, 0 (REF)	619 (71)	252 (29)				
20–24, ≥1 (REF)	4972 (66)	2512 (34)				
20–24, 0	11533 (66)	6051 (34)	1.47 (0.52, 4.12)		1.25 (0.46, 3.38)	
25–29, ≥1 (REF)	4864 (62)	2944 (38)				
25–29, 0	1586 (53)	1392 (47)	2.46 (0.92, 6.53)		1.91 (0.74, 4.90)	
30+, ≥1 (REF)	802 (56)	638 (44)				
30+, 0	197 (41)	282 (59)	4.01 (1.47, 10.93)		3.24 (1.25, 8.41)	
Education (years)						
None (REF)	618 (72)	244 (28)		< .0001		< .0001
1–6	2629 (71)	1087 (29)	1.05 (0.89, 1.23)		1.28 (1.07, 1.54)	
7–12	17072 (64)	9621 (36)	1.43 (1.23, 1.66)		1.50 (1.30, 1.72)	
>12	4268 (58)	3124 (42)	1.85 (1.59, 2.17)		1.89 (1.66, 2.15)	
Gestational Age						
Full term (REF)	22236 (63)	12978 (37)		< .0001		<0.001
Preterm	2351 (68)	1098 (32)	0.80 (0.74, 0.86)		0.82 (0.73, 0.91)	
Birth weight						
Normal (REF)	14831 (62)	9045 (38)		< .0001		<0.01
Low	9756 (66)	5031 (34)	0.85 (0.81, 0.88)		0.91 (0.86, 0.96)	
Anemia						
Yes (REF)	22344 (64)	12433 (36)		< .0001		<0.01
No	2243 (58)	1643 (42)	1.32 (1.23, 1.41)		1.15 (1.05, 1.26)	
Year of Delivery						
2010 (REF)	3196 (72)	1253 (28)		< .0001		< .0001
2011	3236 (70)	1388 (30)	1.09 (1.00, 1.20)		1.07 (0.99, 1.16)	
2012	2980 (67)	1454 (33)	1.24 (1.14, 1.36)		1.22 (1.08, 1.38)	
2013	3300 (66)	1706 (34)	1.32 (1.21, 1.44)		1.28 (1.13, 1.44)	
2014	2963 (63)	1727 (37)	1.49 (1.36, 1.62)		1.46 (1.29, 1.64)	
2015	3047 (58)	2198 (42)	1.84 (1.69, 2.00)		1.76 (1.58, 1.95)	
2016	2972 (57)	2257 (43)	1.94 (1.78, 2.11)		1.81 (1.60, 2.05)	
2017	2893 (58)	2093 (42)	1.85 (1.69, 2.01)		1.71 (1.49, 1.97)	
Year of Delivery Trend Test^c^			1.11 (1.09, 1.13)	< .0001	1.09 (1.08, 1.11)	< .0001

^a^ Includes maternal age, parity, interaction of maternal age and parity, maternal education, gestational age, birth weight, anemia and year of delivery.

^b^ ORs and 95% CI not represented as these variables are part of the interaction term.

c Estimated in a separate model with year of delivery included as a continuous variable.

## Discussion

In the rural and semi-urban population of pregnant women surrounding Nagpur city in Eastern Maharashtra, we found that the odds of having a CS at a private facility were 40% higher than at a public facility after adjusting for other known risk factors. CS rates increased substantially between 2010 and 2017 at both public and private facilities, with privately funded facilities having a higher CS rate throughout the study period. We also observed an unequal spatial distribution of sub-district-based CS rates. CS rates increased more rapidly in the northern and eastern sub-districts during the 8-year study period, perhaps due to mushrooming of more private facilities in these regions.

The odds of having a CS for women in our study increased with more years of maternal education, increasing age and nulliparity, having no maternal anemia and year of delivery and decreased for preterm babies and babies of low birth weight. The same association between having a CS and maternal education has been consistently reported in India [21,22]. Women with more education are likely of higher socioeconomic status and might have resources necessary to access facilities that provide CS and possibly be less likely to tolerate long labor pains, particularly in primiparous mothers. The increased CS rates over time have also been observed elsewhere in India [16,17]. This trend is likely due to the increase in institutional births that occurred following JSY implementation. Maternal anemia is a risk factor for having preterm and low birth weight babies making all three characteristics more likely to result in smaller babies that are easier to deliver vaginally [23].

This study has several strengths. First, this study is a large, pregnancy and newborn, population-based registry that has included standardized, prospectively-collected data since 2009. Second, to our knowledge, this is one of the only studies in India to map the growth of progressively increasing CS rates and attempt to determine drivers of the increase. Finally, over 95% of the pregnant women in the four included districts are enrolled into the registry and follow-up for the study population exceeds 99%. Enrollment of almost all pregnant women in the catchment areas likely helps to reduce selection bias.

This study also has some limitations. First, mapping aggregate data assumes that the data is evenly distributed across both time and the sub-districts, which may not be the case. Second, our subdistrict CS rates were calculated based on where a woman lives, but it is possible that women travel to different subdistricts in order to give birth. More information on individual-level healthcare access is necessary to further explain the increase in CS rates. Third, we did not have data on medical indication for CS for the entire study period (only collected in 2010–2013 as reported in Patel et al [23]). An increase in medical risk factors for CS could explain the increase in CS rate. For this reason, we also could not classify CS as being elective or emergent. Fourth, we did not have information on previous CS and therefore were unable to include this important risk factor in our analysis. While previous CS is an important driver of CS rates, including previous CS rates in our study would only help to understand CS rates in multiparous mothers. Fifth, we did not have information on participant’s socioeconomic status and included maternal education as a proxy. There could be some residual confounding present in our estimates as a result. Finally, we were not able to account for patient preference for type of delivery or amount paid for services as these variables were not available in our dataset, which could create some endogeneity in our independent variable.

## Conclusions

Our study found that CS rates in both private and public facilities in four districts of eastern Maharashtra increased between 2010 and 2017, with rates from women delivering in private facilities consistently higher than those of women delivering in public facilities. A map of the spatial distribution of subdistrict-based CS rates indicates an increase in CS rates more rapidly in northern and eastern subdistricts of the study area. These maps could help both researchers and government officials better understand the patterns of CS deliveries and target future resources appropriately. Further investigation into why these clusters have formed is warranted. Additional research is needed to understand these clusters of rapidly increasing CS rates in order to fully understand patterns of CS deliveries in the study area. Future qualitative studies are required to gather information on reasons for CS in order to formulate policies to reduce unnecessary CS.

## Supporting information

S1 File Data file. (CSV)
